# Then and Now: What Has Caused the Change

**Published:** 1884-12

**Authors:** P. P. Wells


### THEN AND NOW: WHAT HAS CAUSED THE CHANGE?

When I changed my practice from old school to Homœopathy, more than forty years ago, it was accepted by those who had accepted the new therapeutics, that Homœopathy was a law, like other natural laws, of divine origin and authority; that on this law was founded the true and only *science* of therapeutics which truth presented to our confidence, and that the chief duty of healers was to acquaint themselves with this law, its corollaries, and the agents it required for its practical administration, and then to use these agents in clinical duties as the law commands. This, then, it was that constituted the practice of Homœopathy, and the habitual performance of this duty was accepted as constituting a man its *practitioner*. Such practitioners were busy with the study of this law as given and explained only in the *Organon of Homœopathic Medicine*, and with endeavoring to follow as closely as possible the instructions there given for the examination of the sick condition, that the elements by which this is related to its curative might be accurately known, and then to find this curative in their materia medica by reason of the greatest possible similarity in the ascertained effects on the living organism of some *one* of its drugs to these elements. When so found and given, *sicknesses were cured*, and there was a great and bright joy with healers so employed and so successful. The results of such labors were a new revelation to those doctors who had had previous education and experience of "old school physic" only. Law was their master and they obeyed it. They had had no helpers in college or literature to any "short and easy method" of finding their simillimum for each case treated. So they *worked* and found it, as the *Organon* directed, and the result was their great reward—*they cured*. If one had come to these so working, with modern means and appliances for facilitating diagnosis, and through these and thus pointing to a short and easy way to the simillimum, he would most certainly have been set down as a sham. In the *results* of their loyal practice they found no needs for other methods, and then the idea of "labor-saving machine" practice in the interest of the doctor, not of the patient, was not born. The proponent of such ideas would at once have been proclaimed the sham he really was; or, if a homœopathic practitioner, a traitor to the truth he was professing to practice. A *simillimum to diagnosis* had not then been invented. The basis of each prescription then was, as the *Organon* directed, *the totality of the symptoms*, and never a *name*.

Soon after this "The American Institute of Homœopathy" was organized. Its originators were for the most part men who had been thus taught by the *Organon*, and who were thus endeavoring in practice, so far as they were able, to obey its practical precepts. They met and organized in the interests of an increased knowledge of the Homœopathy of the *Organon*, increase of curing agents and of knowledge of those already possessed and used in clinical duties, and to protect the community from impositions of shams and imposters, who might pretend to practice in accord with our law, but who, from ignorance, indolence, or any other cause, were incapable of the practice they pretended to pursue. In the early years of the Institute its members were seen loyally laboring in carrying out these objectives of its creation.

Its activities were great, and its membership increased, and has continued to increase, till now it has become a great multitude, almost wholly composed of successors of its founders, who seem to be occupied and interested in all other branches of medical science, to the great exclusion of the objectives of these founders. Any other science, or *pretense* of science, has their immediate and earnest attention, but for the law, its fundamental rules of practice, its necessary corollaries, its pure materia medica—for these the successors of these founders seem to have little heart or time. They have neither patience nor disposition to listen to any plea for the Homœopathy of their predecessors. When its better practical successes are reported, it is found *easier*, if not wiser, to pooh! pooh! or deny their truth, than to imitate the laborious process which has achieved them, and ease prevails; and the meanness of this is shielded by the cry, "*Don't you see we have the majority on our side?*" If so, then all it proves is that the majority prefer ease to truth, and this can only be shame on the majority. Do these successors forget that the truth is, in itself, always the "majority"? And mere numbers, however great, if opposed to the true and devoted to the false, are only destined to succumb to the one majority in the end. It may be well that this be remembered.

We have said the activities of the founders of our Institute were great. It may be added, that of their successors is scarcely less so. But how great the difference of the objectives on which they have expended their energy?—the one endeavoring to build up and establish a knowledge of truth and confidence in it, the other most intent on destroying both.

Why has this change come upon us? Certainly not because of failure to cure the sick by the earlier, and, we may add, the legal and better method advocated and practiced by those who created this Institute, that extended knowledge of this method might give increased confidence in its truth and efficacy to doctors and men; not because any man of average intelligence who had been honestly endeavoring to obey our law in his practical duties had found any need of the change, nor because any such man has found in the change any better practical results from abandoning law and following traditions of men, old or new. And then, by what agency or authority has this change been effected? We believe we do it no injustice if in tracing its origin and pedigree we find its father to have been *Ignorance,* its mother *Indolence,* and the accoucheur in attendance at its birth, *Conceit.* The result is certainly worthy of this tripartite combination, and seems to bear birth-marks on its front testifying abundantly to its parentage, and in its now early life has shown ample evidence of its ready absorption of the spirit and manners of its nurse—*Impudence.*

But, it may be asked, where is the evidence that this so great change really exists? And it is right the question should be answered.

The first evidences of this change we shall give is, remembering the fact of the early devotion and loyalty of our Institute to law (it has been claimed that this be accepted as our great representative body), we place in sharp contrast with this the notorious "*last resolution,*" passed by this body at Indianapolis, 1882, by which it absolves its membership from all obligation to obey law;—that law which had been accepted as the only formula of a *science* of therapeutics; that law declared, and before believed to be given, of God; that law to which all members of this "representative body" were supposed to have declared allegiance, thus abrogating, so far as it had power, God's law, and making every man a law to himself. Is not this evidence of change in this so-called "representative body"? Is it not clear that, whatever it represents, it has ceased to represent the Homœopathy of its founders? These were willing to acknowledge God to be wiser than men; that He knew and had given to the knowledge of men a *law* to be recognized and obeyed in the use of means for the cure of the sickness of mankind. Not so their successors. To them any man's whim is to be a higher authority than any law of God or man. Is the change for the better? Verily, here is a change, but it is a change "*with a vengeance!*"—a "*progress*" downward and backward.

Second. The Institute in its early history was employed chiefly and earnestly with the interests of its materia medica, to enlarge its record and lessen the difficulties of its practical application; and also with its therapeutics, that by a right understanding of law and its corollaries more perfect successes in healing might be attained. Now the members seem to be more interested in anything else, especially if it be presented with a seeming or claim of the "scientific." This appears from the greater number of bureaus or committees appointed to consider and present other subjects than these specialties of Homœopathy, as compared to the few who were expected to present papers referring to these last, and this is further confirmed by a comparison of the number of papers presented, read, and discussed on other subjects, as compared with the few related to materia medica and therapeutics.

Then, thirdly, the action of the Institute can hardly be accepted as friendly to the best interests of our materia medica in continuing from year to year as Chairman of the bureau of this important science a member who has done nothing for its enlargement, elucidation, or purification. He has done nothing to advance any man's knowledge, or to help his mastery of this important science. He has done nothing, so far as we know, to show he has any especial fitness for the discharge of the important functions which should pertain to this office. He has done nothing, that we are aware of, which he or his friends can present as a reason for his continuance in this position year after year by the presiding officer of the Institute. He has done nothing but discharge the duties of a "common scold" for this honorable body, and in these it cannot be denied he has shown no common diligence. But it may be questioned whether these duties are in themselves or their results so meritorious as to deserve from the body he has so served any very exalted honors or trusts. And this will be the more questionable when it is remembered that the objective of his habitual scolding has been to destroy confidence, so far as he had power, of doctors and patients and all men in the purity of our record of our materia medica. That he has done no more mischief in this matter has been solely because he had no more power. No man will deny he has done all he could. It certainly is not easy to accept this service as evidence of any great or especial knowledge of our materia medica, and the more so as every utterance of his on this subject has been only additional evidence that he knows very little. And now we think we may be warranted in saying that of this kind of evidence we have had fully enough and need no more.

Would the early Institute have honored any man with office for such reason?*

Then next, in matters which have of late interested our Institute most, have been efforts of another kind to sap our confidence in the worth and truth of our materia medica record. In giving countenance and approval to these, the late history of the Institute is in sharp contrast with its earlier, when all were only intent on making this more complete, better known, and more carefully used for curing the sick as directed by our law. We refer to the reported efforts to *see* that which cures in our medicines, and which failing to *see* has been more or less accepted as proof of absence of curing power in the doses of them used. The microscope does not enable one to *see* this power, and therefore it has no existence. This has been the experience and the logic which has been most acceptable to these successors of the founders of this organization. It does not matter that these preparations, where no curing agent can be seen with the help of the microscope, have cured multitudes of grave sicknesses, and cured them in greater proportion and in less time than those where something supposed to be the curing agent can be seen by this help. "It can't be *seen*, and therefore it is not," is the conclusion which has had the approval of these successors of a better generation. The microscopist can't *see* the power, and therefore his logic compels him to discredit uniform clinical testimony to the presence of this power in healings innumerable. "I can't *see* the power, and therefore the alleged cures are only so many practical im-

---

* This persistent and almost perpetual scolding of our materia medica reminds me of a story told by Rev. Henry Ward Beecher in a Presidential canvass not many years ago. The candidate of one side had been attacked by a base and foul slander by the papers and stumpers of the other. This they continued to repeat after it had been abundantly proved to be false. This conduct on their part reminded Mr. Beecher of an Ohio farmer and his large Newfoundland dog. A lively little squirrel often visited the farmer's premises, and as often was attacked by his huge enemy, the dog. The squirrel each time sought and found safety in a hole in a stone wall. In it he was safe beyond the reach of the dog, who, notwithstanding, would bark at the hole a long time after each chase, just as if it did him good and hurt the squirrel a good deal. He became so fond of this that when he saw a traveler coming down the road he would rush for the hole and set up a furious din. His owner one day, after such a display of this habit, reproved his folly. The dog replied by a look, which was construed thus by his master: "Master, I know there is no squirrel there as well as you do, but then you must pardon something to the *nature* of a *dog*, you know." It is possible the *barkers* at our materia medica really believe there is some kind of a squirrel in the hole at which they have disported themselves so long. Is it this or is it their *nature* which prompts their barking with a force they cannot resist? The dog, the wiser animal of the two, knew there was no squirrel.

possibilities." The sick who appeared to be cured, and who thought they were cured, were not cured at all, and it is a sufficient proof of this that even with my microscope I can't *see* what cured them. Let the logic of this philosopher carry him one step further, and let him be consistent, as all good philosophers should be, and deny the existence of *pain* because, forsooth, even with the highest and best powers of his microscope, he cannot *see* it. Experiments of this sort please these successors and they give them their approval. This search for the invisible may have a place in some form of Pickwickian science; with true Homœopathy it has no place. The experiences of true homœopathists abundantly prove that pains and sicknesses can *see* curing power where even the microscope cannot.

But scolding and the microscope have powerful aids in their efforts to destroy confidence in our record of materia medica and therapeutics in the crucible and test-tube. Chemistry joins ambition and the microscope in their endeavors to destroy confidence, and in this goes for "Fincke's high potencies!" It burns them and then goes to poking in the resulting ashes for something which Fincke never potentized, and for his hundred-thousandth potency as well. And why not as well? If the existence of curing power be denied because the microscope cannot *see* it, why not deny its existence because it could not be found by poking in these dead ashes or by any, whatever, resort to the test-tube? The one is certainly as logical as the other, or both are equally powerful or powerless to set aside the therapeutic record of the efficacy of the agents the best clinical administration of Homœopathy has employed. And yet it was a report of such chemical fooling that seemed greatly to interest and excite to an uncommon degree these successors of the founders of the Institute. They acted as if they expected some new birth of greater value to themselves and the rest of mankind than was the older birth of the similars, with which they seemed to be having so little to do and to have had so little of practical knowledge or concern, and that greater good was to come from these quasi-*scientific* foolings than from any knowledge of or obedience to God's given law, so heartily accepted by their more enlightened predecessors. They seem to have accepted these as most "excellent foolings," and were each, more than the other, eager to thrust his hand into his pocket and bring out and contribute the means necessary to their continuance.

But these successors have just closed their session for 1884, the programme for which has recently passed under our review. Of its acts at this session we know but little, and this little, we are sorry to say, is not charged with evidence of improvement in the work performed or proposed as compared with that of its immediate predecessors. Destructiveness rather than development of any truth or interest pertaining to Homœopathy seems to be the genuine characteristic of so much of this as has come to our knowledge. Of this we shall only refer to one matter, of no little importance, and to their proposed method of dealing with it. The "reformed" or "model" materia medica had the attention of these successors, and they assented to a request from English physicians to join them in the labor of its production. This might be a noble work if in the hands of competent and trustworthy men, but when the co-operator assigned by the Institute to join our English workers is no other than its "common scold," as we are already in possession of his notions of "reform" of this work, we are constrained to fear, if his English associates are in harmony with him in these, there is for us little of hope of good in the results. The first objective to which this joint commission give attention seems to be, there is *too much* of our materia medica, and therefore the first idea of its "reform" is *reduction.* But how reduction? Why, by *striking out* "all which is not essential"! Who is to decide this question of essentiality? and by what rule is he to be governed in his decisions? If he err in this and reject a truth (he *may* mistake and do this), who is to be a gainer? and what are we to think of such a reform? But we are in possession of two rules which it may not unreasonably be supposed will be authoritative. The first was given by the American associate in this work that is to be, and is found in his report to the Institute in 1883. The record of no drug is to exceed "five pages." If there be more, this is evidence of "non-essential" character and a sufficient warrant for "striking out" till the regulation size of the record is reached. But what part of this excess is to be sacrificed? and why? For what other reason than this of "too much of it" is this or that to be excluded rather than other facts of the record? And here comes in the second rule with partial relief, furnished by the Institute itself—and we are constrained to say no other of its recent acts surpass this in unmixed silliness, not even the notorious "last resolution." It is this: No symptom is to be accepted which has resulted from any dose of any potentized drug above the tenth number. The importance of the symptom, its truth, its often verification in clinical experience, nor any other facts pertaining to it, are to have any weight with these reformers. They are directed by the Institute to have regard only to the dose from which it is supposed to have come. Verily the length of the ears of the proposer of this rule should be investigated; they must be something phenomenal in extent. The founders of the Institute were just weak enough to believe that the value of a symptom was determined by its truth or falsehood. Not so their successors. They have discovered it is all *in the dose*, and all from above the tenth potence are to go, like Domine Sampson's duodecimos, "over the shoulders," as of no account. (See *Guy Mannering.*) It may be a comfort if we are permitted to hope that silliness has here reached its lowest depth.

P. P. Wells.

